# Identifying Risk Factors for Secondary Infection Post-SARS-CoV-2 Infection in Patients With Severe and Critical COVID-19

**DOI:** 10.3389/fimmu.2021.715023

**Published:** 2021-09-30

**Authors:** Mingquan Guo, Menglu Gao, Jing Gao, Tengfei Zhang, Xin Jin, Jian Fan, Qianying Wang, Xin Li, Jian Chen, Zhaoqin Zhu

**Affiliations:** ^1^ Department of Laboratory Medicine, Shanghai Public Health Clinical Center, Fudan University, Shanghai, China; ^2^ Shanghai Institute of Phage, Shanghai Public Health Clinical Center, Fudan University, Shanghai, China; ^3^ Department of Clinical Laboratory, Obstetrics and Gynecology Hospital of Fudan University, Shanghai, China; ^4^ Department of Thoracic Surgery, Shanghai Pulmonary Hospital, Tongji University School of Medicine, Shanghai, China

**Keywords:** SARS-CoV-2, secondary infection, immune response, metagenomics, risk factors

## Abstract

Emerging evidence has unveiled the secondary infection as one of the mortal causes of post-SARS-CoV-2 infection, but the factors related to secondary bacterial or fungi infection remains largely unexplored. We here systematically investigated the factors that might contribute to secondary infection. By clinical examination index analysis of patients, combined with the integrative analysis with RNA-seq analysis in the peripheral blood mononuclear cell isolated shortly from initial infection, this study showed that the antibiotic catabolic process and myeloid cell homeostasis were activated while the T-cell response were relatively repressed in those with the risk of secondary infection. Further monitoring analysis of immune cell and liver injury analysis showed that the risk of secondary infection was accompanied by severe lymphocytopenia at the intermediate and late stages and liver injury at the early stages of SARS-CoV-2. Moreover, the metagenomics analysis of bronchoalveolar lavage fluid and the microbial culture analysis, to some extent, showed that the severe pneumonia-related bacteria have already existed in the initial infection.

## Introduction

Severe acute respiratory syndrome coronavirus 2 (SARS-CoV-2), a kind of beta coronavirus that infects the host cell *via* its interaction with the angiotensin-converting enzyme–related carboxypeptidase (ACE2), has become the greatest scourge for humanity since the 1918 influenza (1, 2). Unlike other viruses caused severe acute respiratory syndrome, as SARS-CoV-2’s targeted receptor ACE2 is mainly expressed in the epithelial cells of the cardiopulmonary, SARS-CoV-2 infection could lead to dysfunction of alveolar epithelial cells and acute inflammation response, exerting severe acute respiratory syndrome (3). Compared to the SARS and the Middle East Respiratory Syndrome Coronavirus (MERS), the virulence of the SARS-CoV-2 is relatively low, only a small portion of infected people developed into severe and critical syndromes (4, 5). When taking the extremely highly spreading ability of this virus into consideration, in particular the discovery of the new SARS-CoV-2 variant “ (VoC) 202012/01” in the UK and “B.1.617” in India, a greater number of secondary infections and deaths is inevitably accompanied by the enhanced version of the virus (6, 7). Thus, a better understanding of the causes of severe/critical secondary infections and death is urgently needed.

Many efforts have been devoted to unveiling the life-threatening causes of COVID-19, one of the major causes is the secondary infection in those severe or critical patients (8–13). Secondary infection, also known as co-infection, belongs to one of the leading causes of virus-related mortalities, especially the respiratory infections such as viral pneumonia (14, 15). Generally, there are many opportunistic pathogens existing in human body, such as *Mycoplasma pneumoniae, Acinetobacter baumannii*, *Pseudomonas aeruginosa, Haemophilus influenzae*, *Staphylococcus aureus*, *Streptococcus pneumoniae* and other resistant *Enterobacteriaceae* (16–18), with the changes of the immune system and micro-environment when fighting against the viral infection, these opportunists look to expand rapidly and cause severe secondary infection post-SARS-CoV-2-infection (19, 20).

In SARS-CoV-2, many secondary infection events have been observed, including bacterial and fungal infection, especially in those severe*/*critical cases (9–13). The overall secondary infection rate post-SARS-CoV-2 infection is about 3.2% ~ 15%, and the mortality of secondary infection was much higher in those severe and critical COVID-19 cases (8, 9, 21). Still, what caused the secondary pathogens infection remains largely unexplored, and a better understanding of the potential mechanisms underlying secondary infection risk could help us predict the risk of severe disease and take action before it happens.

In this study, we systematically determined the factors contributed to elevated risk factors of secondary SARS-CoV-2 infection in single-center cases from Shanghai Public Health Clinical Center Affiliated to Fudan University. We performed RNA-seq analysis in 43 peripheral blood mononuclear cells (PBMCs) and 5 bronchoalveolar lavage (BAL) samples from COVID-19 patients at initial diagnosis stage. Also, we investigated the correlation between lymphocytopenia, multi-organ damage, coagulation and secondary bacterial infection of SARS-CoV-2. This study will provide prospective evidence of a rational and scientific therapeutic response to the SARS-CoV-2 pandemic and secondary co-infection.

## Materials and Methods

### Subjects and Data Collection

This study was retrospectively conducted in Shanghai Public Health Clinical Center, designated as a designated hospital for COVID-19 treatment in East China, from February to May 2020. Among all the patients, their symptoms and groups were identified strictly in accordance with the novel Coronavirus infection diagnosis and treatment plan (Trial version 7) published by Chinese General Office of the National Health Commission. A total of 458 patients were included in this study. Secondary infection was diagnosed based on the patients’ clinical, radiological and laboratory data. A total of 43 patients including 21 mild/moderate and 22 severe/critical were studied within five days post-symptoms. All the clinical data were recorded in a computerized database in the hospital laboratory medicine department.

### Ethics Statement

This study was approved by the Ethics Committee of Shanghai Public Health Clinical Center (#2020-Y025-01), and informed written consents from all human subjects were signed according to the Declaration of Helsinki. This study was conducted according to the guideline of Novel Coronavirus Laboratory Safety from the General Office of the National Health Commission letter in 2020 (Document No. 70, 2nd edition).

### PBMCs Isolation and BAL Collection

The PBMCs were separated by the Ficoll density gradient centrifugation using the whole peripheral blood samples from remaining samples for clinical examination as previous processing (22). About 4 mL of fresh collected peripheral blood samples in anticoagulant tubes were resuspended with equal volume phosphate buffer saline (PBS), the diluted samples were then gently transferred into a 15 mL tube contains 4mL of Ficoll-paque PLUS (GE Healthcare Life Sciences). The tubes were then centrifugated for 30 min at 400 g, and the PBMCs in the middle layer of the tubes were collected and transferred to a new 15 mL tube (BD Falcon, USA). Then PBMCs were washed twice with PBS and lysed in 1 mL Trizol reagent (Thermo Fisher Scientific, USA). Pulmonary bronchoalveolar lavage fluid was collected and centrifuged for 10 min at 600 g. Then pellets were washed once with PBS and lysed in 1 mL Trizol reagent.

### RNA-seq and RNA-seq Analysis

RNAs were extracted by the Trizol reagent according to the manufacturer’s instructions. The mRNA-seq library was constructed according to the manufacturer’s instructions of the VAHTSTM mRNA-seq V3 Library Prep Kit for Illumina^®^. The libraries were sequenced in the Illumina’s Nova-seq 6000 platform.

The RNA-seq analysis was performed as previously described (23). All reads were mapped to the Homo sapiens (human) genome assembly GRCh38 (hg38) by the STAR software (24). The read counts were extracted by HTseq algorithm (25). Differential expressed genes were analyzed by DESeq2 (26), and genes with two-fold change and adjusted *P*-value < 0.01 were kept.

For an integrative analysis, the expression matrix of PBMC cells in patient with Flu, COVID-19 and healthy donors were obtained from the GEO database under the accession of GSE150728 (27) and GSE149689 (28). The datasets were processed with the Seurat suite (29) and the Harmony algorithm (30).

### Clinical Laboratory Examination and Data Collection

All laboratory tests were conducted in the department of laboratory medicine in the Shanghai Public Health Clinical Center. The blood routine tests, including white blood cell count (WBC), lymphocyte count (LPC), platelet count (PLT), Hypersensitive C-reactive protein (hs-CRP), were analyzed by Blood cell analyzer SYSMEX XN-A100 (Hisense Meikang medical electronics, Shanghai Co., Ltd). The CD3^+^ cell counts, CD4^+^ cell counts, CD8^+^ cell counts, CD19^+^ cell counts, CD16^+^CD56^+^ cell counts, and CD4^+^/CD8^+^ percentage were detected by FACS Canto II (BD Biosciences). Lactate (LACT), lactate dehydrogenase (LDH), Cholinesterase (CHE), Prealbumin (PA), Albumin (ALB) were detected by the Biochemical Immune Automation Analysis Workstation (ARCHITECT 3600J, Abbott Laboratories Co., LTD).

### Microbiology Laboratory Testing

The sputum and bronchoalveolar lavage were used for microbiology laboratory tests. The collected samples were cultured in Columbia Blood Agar plate and chocolate Agar plate (Shanghai Comarja Company) at 35°C, 5% CO_2_, and smear and gram staining were performed simultaneously. The Microflex MALDI-TOF MS mass spectrometry (Brock, Germany) and automatic microbiological analysis system (Biomerieux VITEK231, France) were used for the authentication of the strains of bacteria.

### Quantification and Statistical Analysis

All data in this study were expressed as Mean ± SD as indicated. For parametric analysis, the F test was used to determine the equality of variances between the groups compared; statistical significance across two groups was tested by Student’s t-test, one-way analysis of variance (ANOVA) were used to determine statistically significant differences between multiple groups. Values of *P* < 0.05 were considered to be statistically significant.

## Results

### Demographics, Disease Course, and Outcome of Patients With COVID-19

We studied 458 patients from Shanghai Public Health Clinical Center during January to October 2020. A total of 21 mild/moderate and 22 severe/critical COVID-19 patients were randomly included in this study (**Table 1**). The average age of enrolled patients of the mild/moderate was 59.52 ± 14.36 years, 65.47 ± 13.45 for the severe/critical. Length of hospital stay was 35.23 ± 12.74 *VS* 64.28 ± 22.63. Patients with mild/moderate disease did not resort to respiratory equipment during treatment. In the severe/critical patients, 6 (27.27%) cases were diagnosed with secondary infection. Four patients of severe/critical disease were deceased, all of whom had secondary infection.

**Table 1:** Demographics of patients.

Demographics	Symptoms		*P* Value
	Mild/Moderate (n=21)	Severe/Critical (n=22)	
Age (y, mean ± SD)	59.52 ± 14.36	65.47 ± 13.45	0.131
Sex (male)	11 (52.38%)	14 (63.64%)	
Ventilator	0	7 (31.82%)	
ECMO	0	3 (13.64%)	
Hospital stay (day)	25.23 ± 11.74	54.28 ± 22.63	0.001
Comorbidity			
Renal insufficiency	2 (9.52%)	5 (22.73%)	
Diabetes	1 (4.76%)	6 (27.27%)	
Hypertension	2 (9.52%)	5 (22.73%)	
Respiratory insufficiency	1 (4.76%)	4 (18.18%)	
Malignancy	1 (4.76%)	2 (9.09%)	
Secondary infection	0	6 (27.27)	
Deceased	0	4	

ECMO, Extracorporeal Membrane Oxygenation.

### Abnormal Anti-Viral Response in Severe and Critical SARS-CoV-2

**Figure 1:**
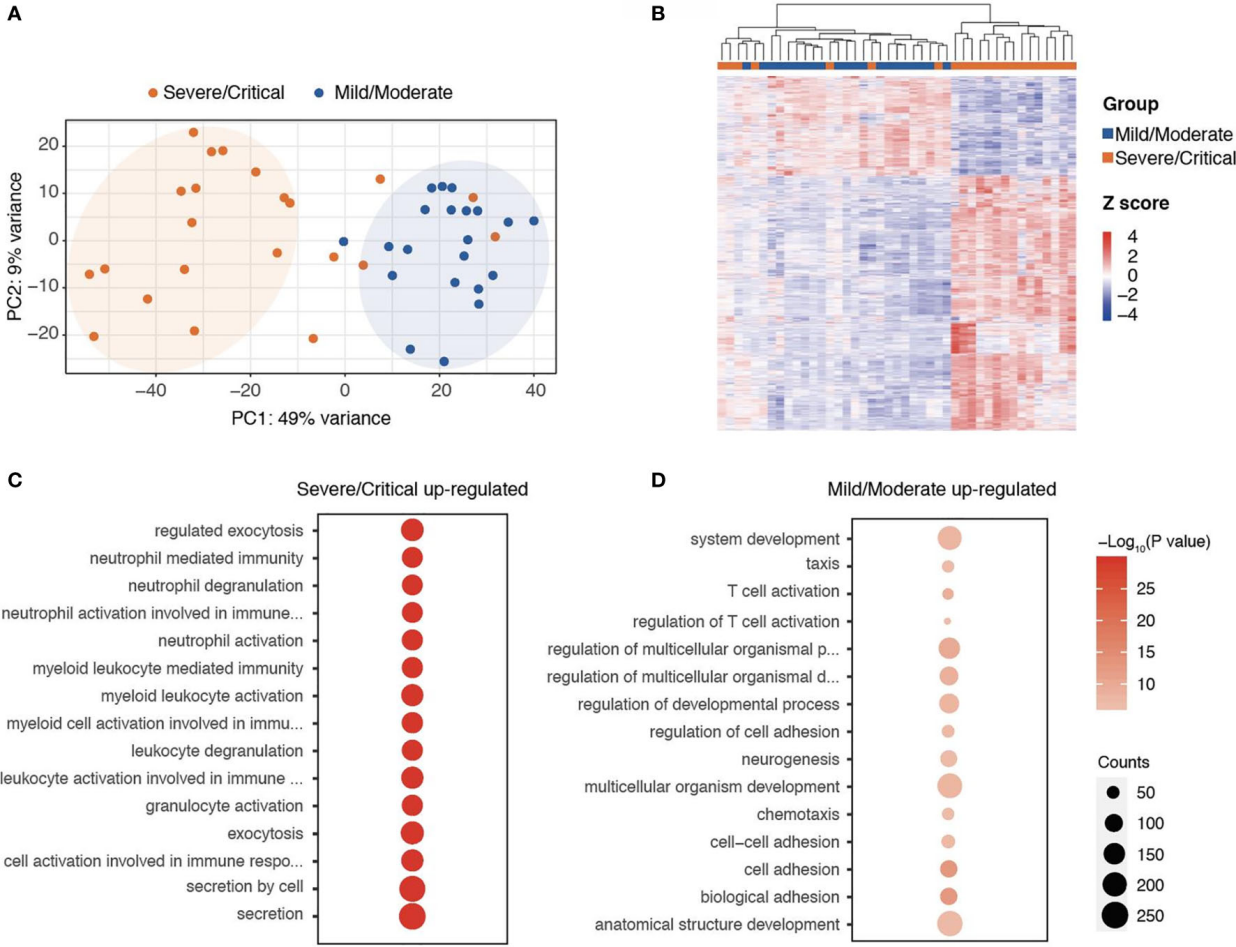
RNA-seq analysis showing the transcriptional programs in blood cells related to the severity of COVID-19. **(A)** PCA analyses of the transcriptome of PBMC cells in mild/moderate and severe/critical COVID-19. **(B)** Heatmap showing the differentially expressed genes in PBMC cells between mild/moderate and severe/critical COVID-19. **(C, D)** Functional annotation of genes correlated with the risk of secondary infection during the initial infection.

To explore the immune response post-SARS-CoV-2 infection, we collected the PBMCs from 21 mild/moderate and 22 severe/critical COVID-19 patients within five days post-symptoms and performed RNA-seq experiments with these collected samples. Gene expression pattern was analyzed to determine the range associated with the disease. Among these 22 severe/critical cases, six developed secondary infection post-SARS-CoV-2 infection. The principal component analysis showing the mild/moderate and severe/critical could be separated according to the expression patterns, indicating the mild/moderate and severe/critical COVID-19 have distinct gene expression signatures (**Figure 1A**). To disclose the distinct transcriptional regulation programs in mild/moderate and severe/critical cases, we analyzed the differentially expressed genes in PBMC cells between mild/moderate and severe/critical samples. A total of 523 genes were significantly highly expressed in mild/moderate and 1,341 genes were significantly highly expressed in severe/critical COVID-19 (**Figure 1B** and **Table S1**).

Functional annotation of these genes demonstrated that genes related to T cell activation, regulation of the developmental process, and chemotaxis were enriched in those genes highly expressed in mild/moderate cases, indicating an active lymphocytic response to viral infections in these mild/moderate COVID-19 patients (**Figure 1C** and **Table S2**). Unlike those mild/moderate cases, we noticed that the severe/critical COVID-19 significantly enriched at genes related to neutrophil degranulation, myeloid leukocyte mediated immunity, and exocytosis (**Figure 1D** and **Table S2**), implying a different response to viral infection in these severe/critical patients as compared to those mild/moderate cases.

### Distinct Expression Signatures of the Risk of Secondary Infection

**Figure 2:**
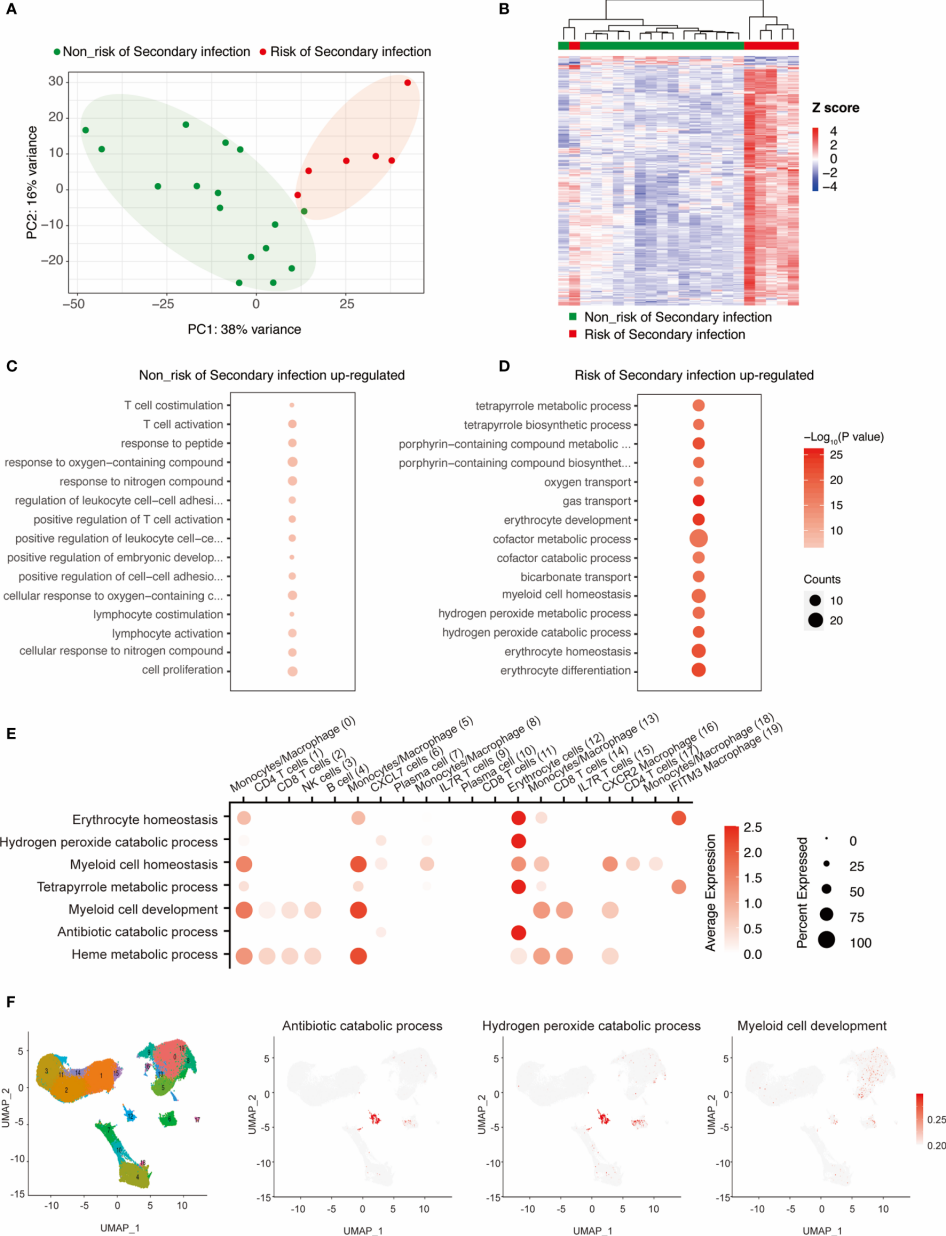
Identifying the key transcriptional programs that might enhance the risk of secondary infection at the initial infection of SARS-COV-2. **(A)** PCA analyses of the severe cases with or without the risk of secondary infection. **(B)** Heatmap showing the differentially expressed genes in PBMC cells between risk and non-risk of secondary infection. **(C, D)** Functional annotation of genes correlated with risk of secondary infection during the initial infection. **(E, F)** scRNA-seq analyses showing the enriched of these risk genes in distinct immune cells from PBMC.

As the secondary infection is one of the leading causes of death, we aim to find some predictive factors associated the potential secondary infection after the initial SARS-CoV-2 infection for the 22 severe/critical cases. We noticed that the transcriptome pattern of cases with the risk of secondary infection was significantly different from those without these risks in the samples of initial infection (**Figure 2A**), highlighting the potential risk had occurred during the initial infection of different individuals. To explore the risk of secondary infection at the initial SARS-CoV-2 infection, we obtained the differentially expressed genes in those cases with or without secondary infection risk. A total of 291 risk-related genes and 11 non-risk related genes were identified (**Figure 2B** and **Table S3**).

Functional annotation analysis of these genes showed that genes associated with T cell activation and lymphocyte co-stimulation were enriched within the non-risk related genes (**Figure 2C**), implying the functional T cell response might protect the severe/critical COVID-19 from secondary infection. Notably, genes involved in the regulation of myeloid cell homeostasis, oxygen transport, erythrocyte differentiation, and antibiotic catabolic process were correlated with an enhanced risk of secondary infection (**Figure 2D** and **Table S4**). Also, the activation of the antibiotic catabolic process was observed in these patients with secondary infection risk. These findings suggest that further monitoring of antibiotic metabolism might be required.

To further explore the cells in the PBMCs contributed to these transcriptional regulatory programs, we retrieved two independent RNA-seq datasets in PBMCs isolated from COVID-19, Flu, and healthy donors (**Figure S1**). And then, we analyzed the enrichment of these genes related to erythrocyte homeostasis, hydrogen peroxide catabolic process, myeloid cell homeostasis, tetrapyrrole metabolic process, myeloid cell development, antibiotic catabolic process, and heme metabolic process in distinct subtypes of cells in PBMCs (**Figures 2E, F**). We noticed that the majority of these processes were enriched in the erythrocyte cells and macrophage cells, indicating the secondary infection risk related to dysregulation of the transcriptional programs was mainly occurring within the erythrocyte cells and macrophage cells.

### Secondary Infection Risk Was Accompanied by Lymphocytopenia

**Figure 3:**
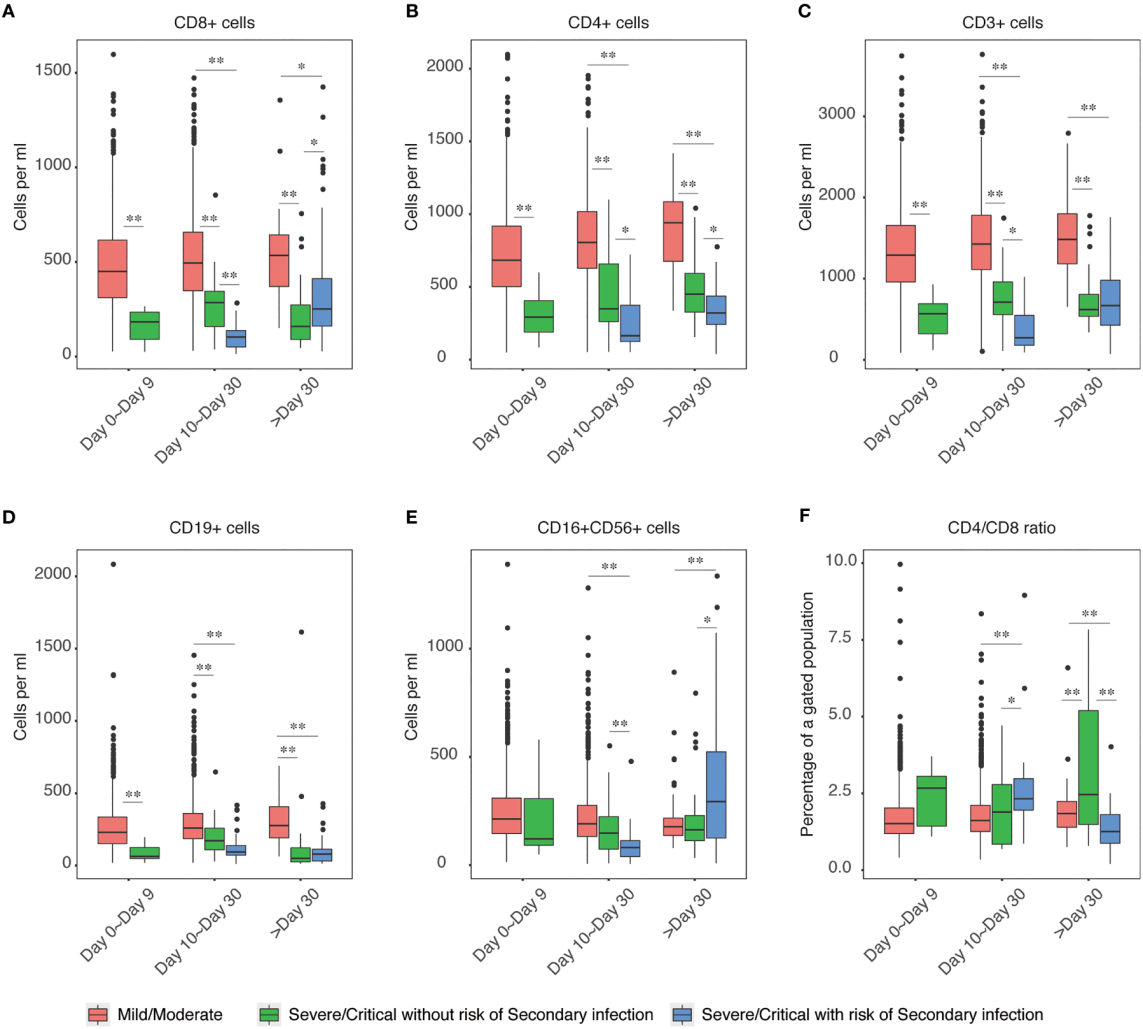
Elevation of lymphocytopenia enhanced the risk of secondary infection. The levels of CD4+ **(A)**, CD8+ **(B)**, CD3+ **(C)**, CD19+ **(D)**, CD16+CD56+ **(E)** and CD4+/CD8+ (F) ratio in mild/moderate COVID-19, severe/critical COVID-19 without risk of secondary infection and severe/critical COVID-19 with the risk of secondary infection were shown. Each group of samples was further divided into three groups according to the time post-symptoms, including day 0 to day 9, day 10 to day 30, and post day 30. The barplot patients with the risk of secondary infection at day 0 to day 9 were not shown due to no available data. The chosen level of statistically significance were *P < 0.01 and **P < 0.05, respectively.

After the initial infection, the immune response was dynamically changed at different stages. We next traced the changes of immune cells in the initial infection stage, intermediate or later stage, and the potential convalescent stages and analyzed the distinct patterns in those patients with secondary infection risks. We found that CD4^+^, CD8^+^, CD19^+^, CD3^+^, and CD16^+^CD56^+^ cells in severe and critical cases were significantly lower than those in mild or moderate cases. Moreover, the levels of these cells were even significantly lower in cases with secondary infection risk as compared to those non-secondary risk severe and critical cases at the intermediate or later stage of COVID-19 infection (**Figures 3A–F**), indicating the secondary infection risk were accomplished with dysfunction of lymphoid cell response, which could also be supported by the genomic response of patients from RNA-seq analyses in **Figure 2B**.

### Liver Injury Is Associated With the Risk of Secondary Infection

**Figure 4:**
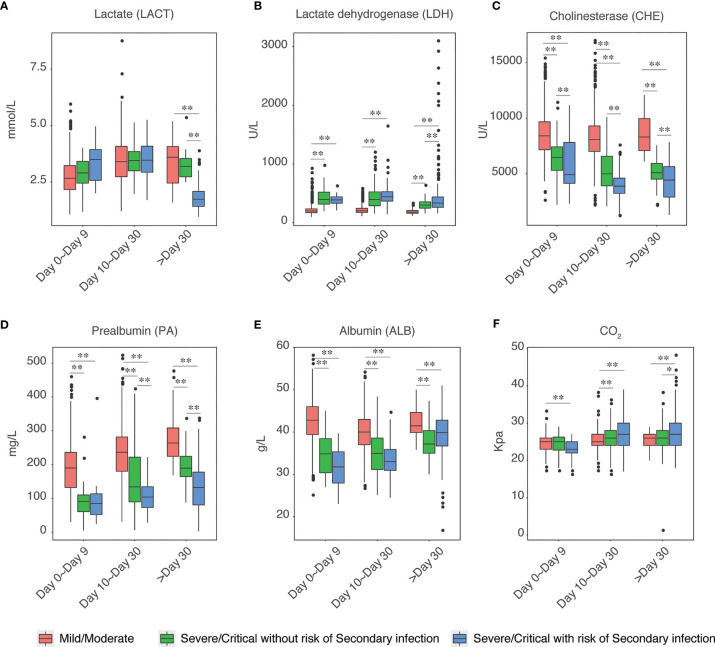
Elevation of Liver injury enhanced the risk of secondary infection. The levels of Lactate **(A)**, Lactate dehydrogenase **(B)**, Cholinesterase **(C)**, Prealbumin **(D)**, Albumin **(E)**, and CO_2_ **(F)** in mild/moderate COVID-19, severe/critical COVID-19 without risk of secondary infection and severe/critical COVID-19 with the risk of secondary infection were shown. Each group of samples was further divided into three groups according to the time post-symptoms, including day 0 to day 9, day 10 to day 30, and post day 30. The chosen level of statistically significance were *P < 0.01 and **P < 0.05, respectively.

Next, we explored the biochemical features in those secondary infection risk and non-risk cases. We found that liver injury-related factors were highly correlated with secondary infection. The levels of lactate, lactate dehydrogenase was significantly higher in severe/critical cases as compared to those mild/moderate cases, and the levels of these two factors were even higher in the cases with secondary infection risk at the initial infection stage (Day 0 to Day 9) (**Figures 4A, B**). Meanwhile, the levels of cholinesterase, pre-albumin, and albumin were significantly decreased in severe and critical COVID-19, and the levels of these three factors were even lower in the severe and critical cases with secondary infection risk at both the initial infection stage and intermediate or later stage (**Figures 4C–F**). Factors associated with kidney injury were not correlated with secondary infection risk (Data not shown).

### Initial Microbial Environment Affected the Risk of Secondary Bacterial or Fungal Infection

**Figure 5:**
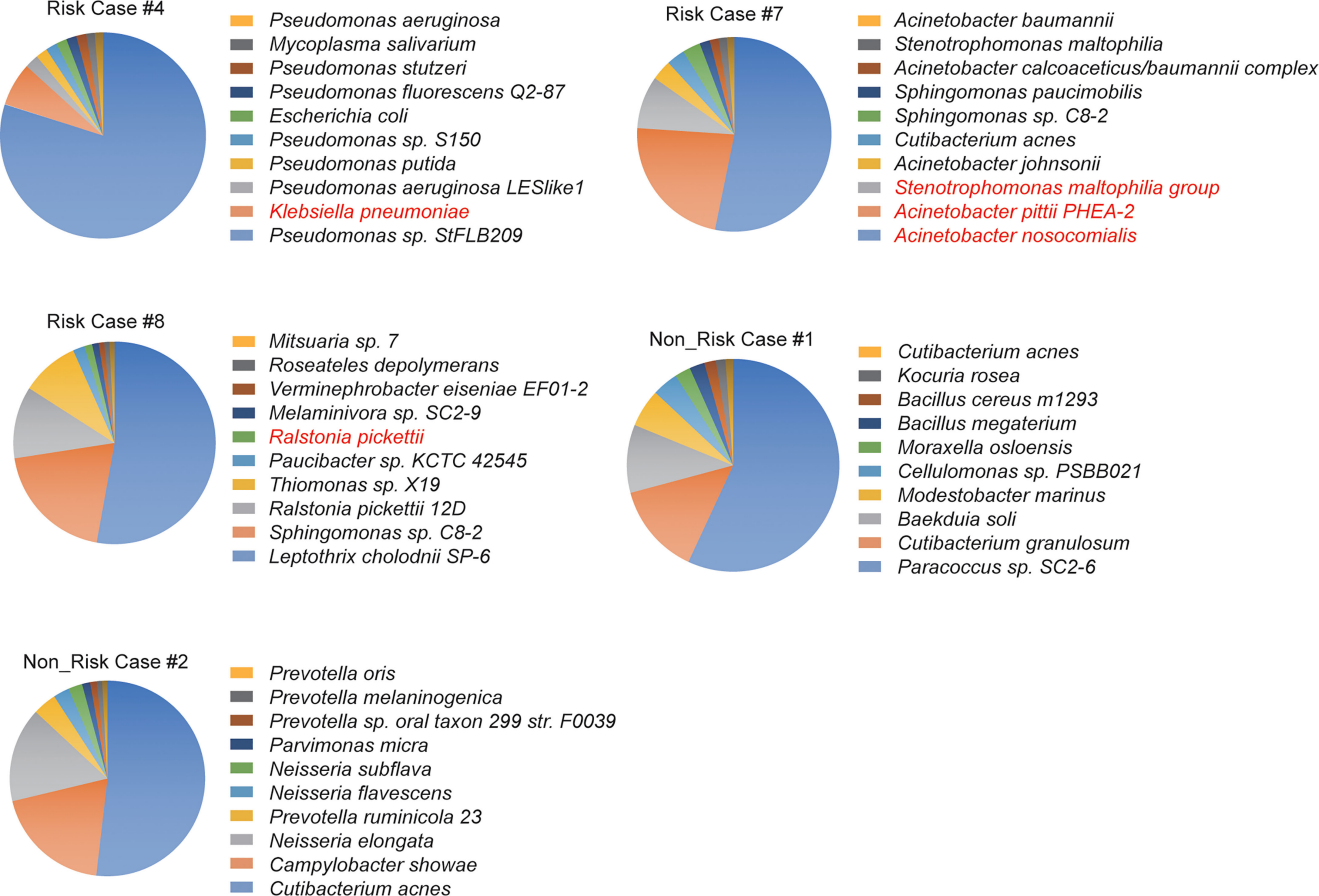
Metagenomic analysis of BALF samples in the initial infection. The top 10 enriched bacteria or fungi were plotted. RNA-seq was performed with the BALF sample in patients with severe/critical COVID-19. All of these patients did not undergo secondary infection yet. All pneumonia-related bacteria were highlighted in red color. Three cases with secondary infection and two cases without secondary infection were included in this analysis.

Next, we explored the correlation between the initial microbial environment and the secondary infection risks. We performed RNA-seq analyses in five bronchoalveolar lavage fluid (BLF) samples isolated shortly after the initial infection according to a clinical report from the Microbiology laboratory, including three developed into a secondary bacterial infection, and analyzed the metagenomic features in these samples (**Table S5**). Many bacteria related to the risk of severe pneumonia were found during the initial infection, including *Klebsiella pneumonia*, *Stenotrophomonas maltophilia*, *Acinetobacter pittii* PHEA-2, *Acinetobacter nosocomialis* and *Ralstonia pickettii* were found in the initial infected samples of COVID-19 patients who developed the secondary bacterial infection, despite these bacteria were not the major bacteria in its environment (**Figure 5**).

Further analysis about bacteria culture results could support these findings (**Table 2**). Of these SARS-CoV-2 patients with co-pathogens infection, the pulmonary infection was the main infection. A few co-pathogens infections occurred in the period after admission, the majority of cases in this study showed sputum/BLF culture-negative for pathogenic bacteria in the initial infection, but the bacteria infection became culture-positive post-SARS-CoV-2 infection for quite a long time. Through the microbial culture results, it is speculated that, to some extent, the secondary infection in some patients may be their own endogenous infection, because our sequencing results in cases 4, 7, 8 were matched with their positive results in the later culture. The dysregulated immune micro-environment, micro-ecology and liver injury provided the chance for expanding the conditional pathogenic bacteria and led to a severe secondary infection.

**Table 2:** General microbiological test information of patients with secondary infection post-SARS-COV-2 infection.

Case ID	Gender	Age	Sample type	Days post symptoms	Bacteria culture results
#1	Male	40	Sputum	3	*Klebsiella pneumoniae*
#2	Male	65	Sputum	22	*Ralstonia mannitole*
			Sputum	22	*Candida albicans*
			Sputum	46	*Acinetobacter baumannii*
			Sputum	46	*Candida albicans*
#3	Male	66	Sputum	7	*Acinetobacter baumannii*
			Sputum	7	*Candida albicans*
			Sputum	30	*Acinetobacter baumannii*
			Sputum	30	*Klebsiella pneumoniae*
#4	Male	76	Sputum	27	*Aspergillus flavus*
#5	Male	82	Sputum	49	*Klebsiella pneumoniae*
#6	Male	63	Urine	74	*Klebsiella pneumoniae*
			Sputum	74	*Acinetobacter baumannii*
			Sputum	74	*Klebsiella pneumoniae*
#7	Male	83	Sputum	48	*Klebsiella pneumoniae*
			Sputum	65	*Aspergillus flavus*
			Sputum	65	*Enterococcus faecium*
#8	Male	79	Sputum	13	*Klebsiella pneumoniae*
			Sputum	18	*Klebsiella pneumoniae*
			Sputum	18	*Enterococcus faecium*
			Sputum	18	*Candida albicans*
			Sputum	30	*Acinetobacter baumannii*
			Sputum	35	*Acinetobacter baumannii*
			Sputum	35	*Klebsiella pneumoniae*
			Sputum	44	*Acinetobacter baumannii*
			Sputum	44	*Klebsiella pneumoniae*
			Sputum	51	*Acinetobacter baumannii*
			Sputum	51	*Klebsiella pneumoniae*
			Sputum	53	*Acinetobacter baumannii*
			Sputum	53	*Klebsiella pneumoniae*
			Sputum	78	*Acinetobacter baumannii*
			Sputum	78	*Klebsiella pneumoniae*
			Sputum	88	*Acinetobacter baumannii*
			Sputum	88	*Klebsiella pneumoniae*
			Sputum	92	*Acinetobacter baumannii*
			Sputum	92	*Klebsiella pneumoniae*
#9	Female	64	Lavage fluid	44	*Klebsiella pneumoniae*
			Lavage fluid	69	*Klebsiella pneumoniae*
			Sputum	34	*Ralstonia mannitole*
			Sputum	43	*Klebsiella pneumoniae*
			Sputum	68	*Klebsiella pneumoniae*
			Sputum	68	*Ralstonia mannitole*
#10	Male	66	Sputum	27	*Klebsiella pneumoniae*
			Sputum	27	*Stenotrophomonas maltophilia*
			Sputum	44	*Klebsiella pneumoniae*
			Sputum	44	*Stenotrophomonas maltophilia*
			Sputum	64	*Klebsiella pneumoniae*
			Sputum	64	*Stenotrophomonas maltophilia*
			Sputum	72	*Klebsiella pneumoniae*
			Sputum	72	*Stenotrophomonas maltophilia*

## Discussion

In the face of the COVID-19, currently rampaging around the world and continues to spread, the secondary infection has been reported to be an adverse prognostic marker for the severe illness and mortality of SARS-CoV-2 (8, 31). It was documented than the mortality rate of secondary bacterial infections among COVID-19 patients admitted to the intensive care unit (ICU) was as high as 95% (32). Identifying potential predictive markers for secondary infection could help a better precaution, but the risk factors but the risk factors contributing to secondary infection are unclear. There has previously documented that the mechanism of the virus‐bacteria co-infection mainly involves the lack of effective immune response and pathogenic synergism by different factors (33, 34). We here reported that genes related to myeloid cell homeostasis, antibiotic catabolic process, and erythrocyte homeostasis pathways were dysregulated in severe and critical SARS-CoV-2 with the potential risk of secondary infection. Furthermore, we demonstrated that the liver injury-related factors at the early stage of infections, lymphocytopenia risk-related factors at the intermediate and late stages, and severe/critical SARS-CoV-2 with secondary infection risks are more abnormal compared to severe/critical cases with non-secondary infection risks.

Noteworthy, viral-caused pneumonia, such as influenza virus and coronavirus, usually led to an exhausted and fragile immune system due to the decreased immune cells when it fights against the virus (35, 36). Thus, viral infection suppresses certain changes in the body’s immune system, and secondary infection can be a life-threatening factor, especially in virus-induced pneumonia (37). In accordance with previous observation, we observed genes correlated with T cell activation were less activated in those severe/critical COVID-19 compared to the mild/critical patients (3, 38, 39). Moreover, the T cell activation and lymphocyte activation programs in the severe/critical cases with secondary infection risks were less activated than those in non-secondary-infection-risk groups of severe/critical COVID-19. Thus, the initial immunities of changed situation were different, and the T cell immunity was abnormal in severe/critical cases, especially those with potential secondary infection risks (22). These observations could be further verified by the flow cytometry analyses of the subset of lymphocytes, and we found that the CD4^+^ and CD8^+^ T cells were extremely low in patients with risk of secondary infections. In our previous work, we found that the frequencies of total lymphocytes and T cells decreased significantly in acutely infected COVID-19 patients compared to healthy controls (40).

In the transcriptomic analysis in PBMC samples, we found that the antibiotic catabolic process was activated in patients with risks of secondary infection. Since antibiotics are the major therapeutic approaches to bacterial infection, the elevated levels of antibiotics metabolism might lower the levels of antibiotics in the circulation system, which might increase the risk of bacterial and fungal co-infection (41, 42). Still, these speculations should be further validated by monitoring the plasma concentration of the antibiotics post-therapy.

The liver is generally considered as an organ involved in the immune response (43). The liver injury might partially lead to immune system failure in response to viral and bacterial infections (44). We noted that the liver injury-related markers, including decreased pre-albumin, albumin, and cholinesterase levels, were observed at the initial stages, and the intermediate stage of SARS-CoV-2 infection in severe and critical COVID-19. Moreover, the albumin and cholinesterase levels were even lower in those cases with the risk of secondary infection, highlighting a link between liver injury at the initial infection stage and the secondary infections. Many studies in COVID-19 have reported liver injury in severe and critical cases (9, 45). We here noticed that the liver injury happened at the initial stages of COVID-19, and thus it might be used as an indicator for the risk of secondary infections in severe and critical cases.

Similar to other viral infections, emerging evidence suggests that an increasing number of COVID-19 patients are being diagnosed with bacterial co-infections during hospitalization (32, 46). Studies about secondary infection to influenza infection have demonstrated that neuraminidase (NA) of influenza virus can activate transforming growth factor-β (TGF-β) in the host, resulting in increased expression of adhesion molecules on the surface of host cells. It increases bacterial adherence to host lung cells, which causes secondary bacterial infections and predisposes the host to secondary bacterial pneumonia (47). The source and specific nature of these pathogens are yet to be fully explored, but there is some evidence suggesting that multidrug‐resistant bacteria are thought to be responsible for the development of these infections. In one study conducted in Iran, the incidence of *A. baumannii* and *S. aureus* were the most common in the ICU (32). However, given lack of the data about SARS-CoV-2 secondary infection, we investigated the bacterial population in the pulmonary microenvironment yielded the bacterial class of the initial infection, and by comparison with the results of microbial culture after infection, it was found that to a certain extent, the culture-positive bacteria in the infection of some patients may be their own endogenous bacteria, of course, our speculation was based on the evaluation of patients with secondary infection. Further work is required to investigate whether there are other factors associated co-infected with COVID-19.

In summary, increasing co-infection is a non-negligible factor in COVID-19, our study identified some abnormalities in the early stage of SARS-CoV-2 infection that might contribute to secondary bacterial and fungal infections. We emphasized the concern of bacterial infections in COVID-19 patients, this could help us better to prevent worse outcomes due to secondary infection. Also, we provided some predictors for the pre-treatment of antibiotics in those risks of secondary infection cases. Still, there are some limitations to our study, with a small sample size, we only evaluated the peripheral blood immune response and clinical manifestations of patients in different groups, other studies with an expanded sample size might be conducted to address these issues further.

## Data AvailabilityStatement

The data presented in the study are deposited in the GEO database repository, accession number GSE184401.

## Ethics Statement

The studies involving human participants were reviewed and approved by The Ethics Committee of Shanghai Public Health Clinical Center Affiliated to Fudan University (#2020-Y025-01). The patients/participants provided their written informed consent to participate in this study. Written informed consent was obtained from the individual(s) for the publication of any potentially identifiable images or data included in this article.

## Author Contributions

ZZ, MQG, and JC designed the experiments. MG, JG, and MQG performed the majority of experiments. MG and JC performed the bioinformatics analyses. TZ, XJ, and JF collected the clinical data. QYW, XL performed the laboratory detection. MQG wrote the manuscript. All authors contributed to the article and approved the submitted version.

## Funding

This work was supported by grants from the National Natural Science Foundation of China (NSFC, 82101847), the Emergency Project of Shanghai Science and Technology Commission (No. 20411950502), the Shanghai Sailing Program (No. 21YF1438800) and Project of Shanghai Public Health Clinical Center (No. KY-GW-2020-15).

## Conflict of Interest

The authors declare that the research was conducted in the absence of any commercial or financial relationships that could be construed as a potential conflict of interest.

## Publisher’s Note

All claims expressed in this article are solely those of the authors and do not necessarily represent those of their affiliated organizations, or those of the publisher, the editors and the reviewers. Any product that may be evaluated in this article, or claim that may be made by its manufacturer, is not guaranteed or endorsed by the publisher.
