# Corrigendum: Identifying risk factors for secondary infection post-SARS-COV-2 infection in patients with severe and critical COVID-19

**DOI:** 10.3389/fimmu.2024.1548101

**Published:** 2025-01-24

**Authors:** Mingquan Guo, Menglu Gao, Jing Gao, Tengfei Zhang, Xin Jin, Jian Fan, Qianying Wang, Xin Li, Jian Chen, Zhaoqin Zhu

**Affiliations:** ^1^ Department of Laboratory Medicine, Shanghai Public Health Clinical Center, Fudan University, Shanghai, China; ^2^ Shanghai Institute of Phage, Shanghai Public Health Clinical Center, Fudan University, Shanghai, China; ^3^ Department of Clinical Laboratory, Obstetrics and Gynecology Hospital of Fudan University, Shanghai, China; ^4^ Department of Thoracic Surgery, Shanghai Pulmonary Hospital, Tongji University School of Medicine, Shanghai, China

**Keywords:** SARS-CoV-2, secondary infection, immune response, metagenomics, risk factors

In the published article, there was an error in the **Funding** statement. The incorrect grant number for the funding received by the National Natural Science Foundation of China was listed. The correct **Funding** statement appears below.

“This work was supported by grants from the National Natural Science Foundation of China (NSFC, 82101847), the Emergency Project of Shanghai Science and Technology Commission (No. 20411950502), the Shanghai Sailing Program (No. 21YF1438800) and Project of Shanghai Public Health Clinical Center (No. KY-GW-2020-15).”

The authors apologize for this error and state that this does not change the scientific conclusions of the article in any way. The original article has been updated.

