# Supplementary material for: Identifying Risk Factors for Secondary Infection Post-SARS-CoV-2 Infection in Patients With Severe and Critical COVID-19
Source: Front Immunol. 2021 Sep 30;12:715023. doi: 10.3389/fimmu.2021.715023 (PMC8514874; doi:10.3389/fimmu.2021.715023)
Supplement: Supplementary file 1 [file DataSheet_1.docx]

**Supplementary Figure and Figure legends**

**
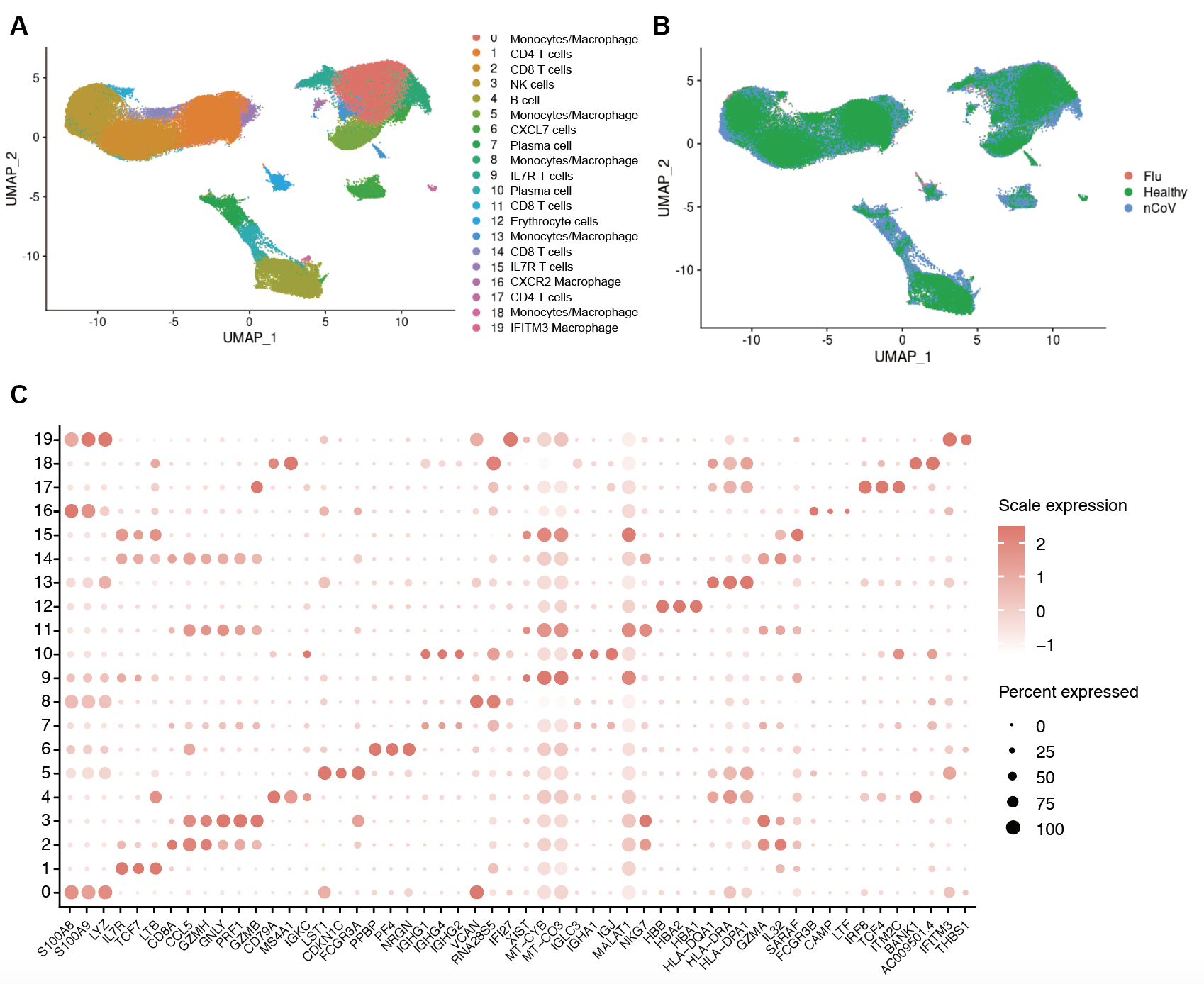
**

**Figure S1. Integrative analysis of scRNA-seq in patients with COVID-19.**

(A) Umap showing the 20 clusters identified in the PBMC samples. (B) Distribution of Flu, COVID-19 and healthy samples in the scRNA-seq analysis. (C) Dotplot showing the representative gene expression among the 20 clusters.
